# Symmetrical organization of proteins under docked synaptic vesicles

**DOI:** 10.1002/1873-3468.13316

**Published:** 2019-01-18

**Authors:** Xia Li, Abhijith Radhakrishnan, Kirill Grushin, Ravikiran Kasula, Arunima Chaudhuri, Sujatha Gomathinayagam, Shyam S. Krishnakumar, Jun Liu, James E. Rothman

**Affiliations:** ^1^ Department of Microbial Pathogenesis Yale University School of Medicine New Haven CT USA; ^2^ Institute of Nautical Medicine Co‐innovation Center of Neuroregeneration Nantong University China; ^3^ Department of Cell Biology Yale University School of Medicine New Haven CT USA; ^4^ Department of Clinical and Experimental Epilepsy UCL Queen Square Institute of Neurology London UK

**Keywords:** cryo‐electron tomography, PC12 cells, regulated exocytosis, SNARE proteins, synaptotagmin

## Abstract

During calcium‐regulated exocytosis, the constitutive fusion machinery is ‘clamped’ in a partially assembled state until synchronously released by calcium. The protein machinery involved in this process is known, but the *supra*‐molecular architecture and underlying mechanisms are unclear. Here, we use cryo‐electron tomography analysis in nerve growth factor‐differentiated neuro‐endocrine (PC12) cells to delineate the organization of the release machinery under the docked vesicles. We find that exactly six exocytosis modules, each likely consisting of a single SNAREpin with its bound Synaptotagmins, Complexin, and Munc18 proteins, are symmetrically arranged at the vesicle–PM interface. Mutational analysis suggests that the symmetrical organization is templated by circular oligomers of Synaptotagmin. The observed arrangement, including its precise radial positioning, is in‐line with the recently proposed ‘buttressed ring hypothesis’.

## Abbreviations

**CLEM,** correlative light‐electron microscopy

**Cpx**, complexin

**EM**, electron microscopy

**ET**, electron tomography

**fLM**, fluorescence light microscopy

**MBIR**, model‐based iterative reconstruction

**NGF**, nerve growth factor

**PM**, plasma membrane

**SNAP**, SNARE protein

**SV**, synaptic vesicles

**Syt**, synaptotagmins

**VPP**, volta phase plate

The release of neurotransmitters stored within synaptic vesicles (SV) occurs in a remarkably synchronous fashion, taking place between 200 μs and 1 ms after the action potential triggered influx of calcium ions (Ca^2+^) 1, 2. The central components involved are the synaptic SNARE (soluble *N*‐ethylmaleimide–sensitive factor attachment protein receptor) proteins that catalyze SV fusion and fusion regulators, complexin (Cpx) and Synaptotagmins (Syt) 1, 2, 3. During this process, partially‐zippered SNARE complexes (SNAREpins) tightly clamped by Cpx and Syt1 are synchronously released to complete fusion 2, 3. Because each individual SNAREpin requires ~ 1 s to drive fusion 4, 5, 6, either very large numbers of SNAREpins would be required, or alternatively a more modest number of SNAREpins would need to be organized in a highly co‐operative framework.

In principle, there could be up to ~ 70 SNAREpins formed because each SV contains about this number of copies of the v‐SNARE, VAMP2/Synaptobrevin 7. But this hypothesis seems untenable because several studies have suggested that synchronous release can still take place with as few as 2–3 copies of SNARE proteins (SNAP) 8, 9, 10. Evidence favoring the main alternative, a well‐organized co‐operative structure, has come from the recent discovery that the Ca^2+^ sensor/clamp Syt1 self‐assembles *in vitro* into ring‐like oligomers based on polymerization of its C2B domains 11, 12, 13. Indeed, a targeted mutation (F349A) in the Syt1 C2B domain that selectively destabilizes the Syt1 oligomers was found to release the fusion clamp and increase spontaneous exocytosis in a model neuroendocrine lineage cell line 14. However, the proposed ring‐like oligomer of Syt1 has never been visualized, directly or indirectly, in cells.

X‐ray crystallography has revealed that two Syt1 molecules simultaneously bind the SNAREpin on opposite surfaces, each interaction mediated by its respective C2B domain 15. One Syt1 C2B domain (‘primary’) binds to SNAP‐25 within the SNAREpin in a manner compatible with its further assembly into a Syt1 ring‐oligomer and likely contacts the plasma membrane (PM). The other Syt1 C2B domain (‘tripartite’) is bound in an incompatible manner, and likely contacts the SV surface^16^. Both the primary and tripartite‐binding sites are required for clamping and for synchronous release 15.

We recently showed how these structural features, namely the Syt1 ring oligomers and the dual‐SNARE binding module, can be easily and elegantly combined with the curved structure of the SNARE‐chaperone Munc13 to form a symmetrical assembly between the SV and PM 16. This proposed organization (‘buttressed ring’) will naturally template a defined number of SNAREpins, sterically block their full‐assembly, and co‐operatively release them upon Ca^2+^‐influx 16.

If this or another *supra*‐molecular organization of SNAREpins were to exist under a docked vesicle, it could potentially be visualized by cryo‐electron tomography (cryo‐ET), which has been successfully used to reveal symmetrical protein structures *in situ* in cells at resolution sufficient to delineate their overall shape and locate protein constituents within them based on high‐resolution structures of isolated components 17, 18, 19.

Here, we report the initial results from cryo‐ET concerning the organization of the clamped exocytic machinery under SVs in neurites that develop from nerve growth factor (NGF)‐differentiated pheochromocytoma (PC12) cells. Consistent with the buttressed ring hypothesis, we observed six distinct masses arranged symmetrically at the SV–PM interface. Each of these rod‐like densities could accommodate the full complement of the minimal release machinery, including a SNAREpin with its two Syt1, a Cpx, and a copy of the other assembly chaperone Munc18. Even though the Syt1 ring‐like structure could not be visualized at this limited resolution, the symmetrical arrangement was entirely disrupted with the Syt1 F349A mutation. In sum, our data strongly suggests that a small number of SNAREpins prearranged by Syt1 ring‐like oligomers represents a central organizing principle of Ca^2+^‐regulated exocytosis.

## Materials and methods

The overall workflow of experimental procedures is outlined in Fig. S1. To generate the VAMP2‐4X‐pHluorin construct, we used a QuickChange mutagenesis kit (Agilent Technologies, Santa Clara, CA, USA) to introduce L70D, A74R, A81D & L84D mutations into the previously described VAMP2‐pHluorin construct 20, 21. The Syt1 F349A used has been described previously 14. The rat adrenal PC12 cell line used was a gift from B. Ehrlich, Yale University.

### Preparation of electron microscopy grids

To allow for *in situ* imaging, the rat adrenal PC12 cells were grown directly on EM grids and grids were prepared for seeding as follows: Quantifoil R2/1 Au‐grids (200 mesh with holey carbon film of 2 μm hole size and 1 μm spacing) or Quantifoil R2/1 + 3 nm C Au‐NH_2_ finder grids (252 mesh with holey carbon film of 2 μm hole size and 1 μm spacing; Electron Microscopy Sciences, Hatfield, PA, USA) were first screened under the light microscope to confirm the carbon film is intact. Three to four grids were placed in the center of a 35 mm glass bottom ‘MatTek’ dish (0.15 mm in thickness and 2 mm in diameter; MatTek Corp., Ashland, MA, USA). The carbon side of the grids were glow discharged for 25 s at 15 mA in a plasma cleaner (PELCO easiGlow, Ted Pella, Redding, CA, USA). The grids were then sterilized in 70% ethanol for 5–10 min under UV, rinsed with distilled water (minimum six times) and incubated for 30 min with 20 μg·mL^−1^ human plasma fibronectin (FC010, Millipore‐Sigma, Burlington, MA, USA) in a 37 °C incubator. The EM grids were subsequently washed with distilled water, and incubated in complete culture medium for at least 6–8 h before seeding the cells onto them. Care was taken during the washes and medium exchange steps, to avoid touching the grids and to prevent them from drying out.

### Cell culture and differentiation

PC12 cells were cultured in Dulbecco's modified Eagle's medium supplemented with 10% normal horse serum, 5% fetal bovine serum, sodium pyruvate, nonessential amino acids and penicillin‐streptomycin and maintained at 37 °C in a 95% humidified incubator with 5% CO_2_ before the experiments. About 24 h before transfection, the medium was replaced with complete medium containing 100 ng·mL^−1^ NGF to differentiate the cells. The NGF‐differentiated PC12 cells were transfected with VAMP2‐4X‐pHluorin (with or without Syt1 F349A) by electroporation (Amaxa; Lonza Bioscience, Allendale, NJ, USA) and the PC12 cells were suspended in a fresh medium containing NGF. The transfection efficiency was typically ~ 60%, with ~ 20‐fold overexpression of VAMP2‐4X over the endogenous VAMP2 in the transfected cells (Fig. S2). About 30 min after transfection, the cell suspension was diluted to a low density (50 000 cells·mL^−1^) and seeded onto EM grids. The cells were monitored under the light microscope every other day for a period of 7‐days for cell confluence and to ensure grid integrity, prior to freezing. All experiments were conducted on PC12 cells cultured for < 10 passages.

### Correlative fluorescence light microscopy

To locate VAMP4X‐pHluorin transfected PC12 cells and neurite varicosities the cells on glass‐bottom MatTek dishes were imaged by fluorescence light microscopy (fLM) at 37 °C in the live cell imaging solution in a Leica DMi8 Wide‐Field microscope 7 days after NGF‐treatment; under conditions cells were ~ 30% confluent. For correlative microscopy imaging, a stitching map of the whole EM grid was acquired at 40× magnification using an automated ‘relative focus controller’, to keep the sample in focus.

### Cell vitrification

Following fLM, BSA‐coated 10 nm Gold Tracer beads (Aurion, Wageningen, the Netherlands) were applied to grids as fiducial markers, immediately prior to vitrification. Grids were then mounted on a home‐made manual plunger, blotted from the back side for 4 s using Whatman #1 filter paper (Sigma‐Aldrich, St. Louis, MO, USA) and rapidly plunge‐frozen into liquid ethane cooled down to a liquid nitrogen temperature.

### Cryo‐electron tomography

The frozen‐hydrated specimens were imaged using a 300 kV Titan Krios transmission electron microscope (Thermo Fisher Scientific, Hillsboro, OR, USA) equipped with a volta phase plate (VPP) and an energy filter mounted in front of a K2 Summit direct electron detector (Gatan, Pleasanton, CA, USA). Images were collected using serialem (Version 3) 22. Initially, a 9 × 10 full‐montage was recorded at low magnification (220×) to produce a complete grid overview and merged with the fLM stitching map. After mapping positions that corresponded to transfected cells, higher magnification (3600×) 2 × 2 montages were acquired to select the suitable positions for cryo‐ET. The tilt‐series images were acquired with the following parameters: 26 000× magnification; tilt range ± 51°; tilt increment 3°; total dose ~ 50 *e* ^−^/Å^2^; pixel size 5.4 Å; and defocus with a phase plate −0.3 μm. The K2 camera was operated in dose fractionation mode. The real‐time phase shift was calculated with gctf software (Version 1.06), and changed to the next VPP when the phase shift is over 135°. Five hundred seventy‐four tomograms were collected from at least 100 cells grown on nine different grids from seven independent cultures.

### Tomogram reconstruction

Tilt series were first aligned using motioncor2 23 and then assembled into the drift‐corrected stacks by tomoauto 24. Subsequently, tomograms were aligned using the imod software package 25, 26, with fiducial markers or fiducial‐free cross correlation, and reconstructed by Simultaneous Iterative Reconstruction Technique 27 or model‐based iterative reconstruction (MBIR) method 28. We used the *XZ* plane and *YZ* plane reconstructed by MBIR method to define the boundary of neurite bouton as this had improved contrast. Furthermore, BSA‐coated fiducial gold beads were utilized to define the top membrane. The PMs and mitochondria membrane structures were manually segmented using imod software package. Microtubules and vesicles, including SV and dense core vesicles, were segmented using eman2 29, 30. UCSF Chimera was used to visualize the tomogram and subtomogram structures in 3‐D and build the atomic model 31.

### Vesicle diameter measurement and statistical analysis

To quantitatively address the proportion and number of SVs differentiated by NGF, we measured the area of vesicles in neurite varicosities and calculated the diameter of these vesicles assuming they are spheres. imod was used to generate TIFF and AVI files and vesicle diameters (in pixels and nm) were measured using imagej software (Version 1.52d) and Excel to carry out the relative frequency statistical analysis.

### Subtomogram analysis

Subtomogram analysis was accomplished from 4757 SVs extracted from 574 tomograms 32, 33. Briefly, we first visually identified the SV with the diameter size smaller than 100 nm. Subtomograms (256 × 256 × 256) of each SV were extracted from original tomograms. Conventional imaging analysis, including 4 × 4 × 4 binning and low‐pass filtering, was used to enhance the contrast of subtomograms. The 4 × 4 × 4 binned subtomograms (64 × 64 × 64 voxels) were used for initial alignment. After an initial alignment based on the global average, multivariate statistical analysis and hierarchical ascendant classification were applied to sort out the vesicles with different sizes. Only ~ 45 nm vesicles were selected for focused classification and alignment at the interface between the vesicle and the PM. After five cycles of rotational and translational alignment with 4 × 4 × 4 binned subtomograms, we used original subtomograms to refine the structure. The CryoEM density map has been deposited in the Electron Microscopy (EM) Data Bank under accession number EMD‐0413 (nonsymmetrized) and EMD‐0414 (symmetrized).

## Results and Discussion

**Figure 1:**
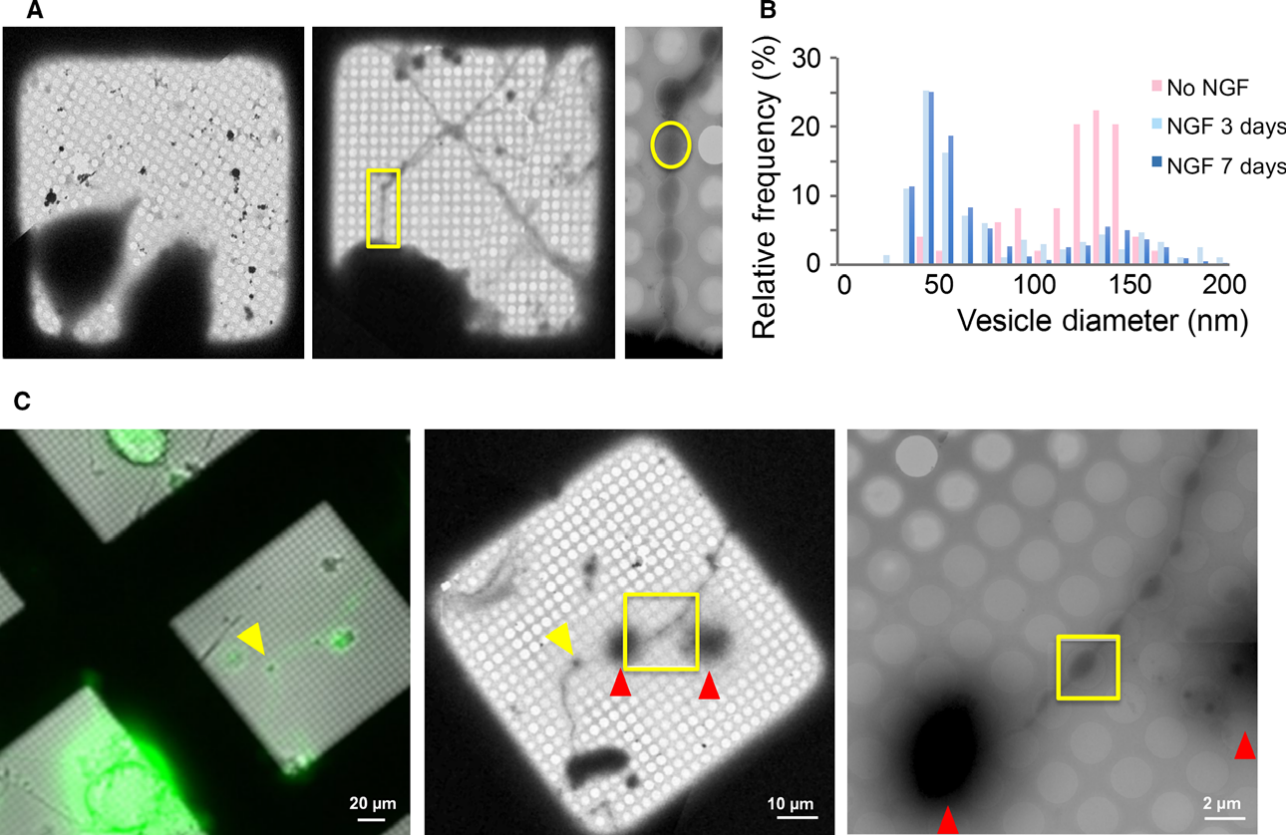
The neurite varicosities of NGF‐differentiated PC12 cell revealed by CLEM and cryo‐EM. (A) Micrographs of PC12 cells grown directly on Au‐grids with or without NGF differentiation, shows the appearance of extended neurite (yellow box in middle plane) with varicosities (yellow circle in the right panel) after NGF differentiation. These neurite varicosities were thin enough to allow cryo‐EM analysis. (B) NGF‐differentiation (3 days or 7 days) increased the relative frequency of small, clear synaptic‐like vesicles as compared to large, dense core vesicles. The vesicle diameters were estimated from tomograms assuming they are spheres. (C) A representative cryo‐CLEM workflow used to identify and image VAMP2‐4X‐pHluorin expressing neurites. Left panel: A fluorescent light microscope and wide bright‐field LM image which were used to identify VAMP2‐4X‐pHluorin‐transfected neurite (yellow arrow). Middle panel: low magnification (220×) cryo‐EM image of the same area. Right panel: high magnification cryo‐EM image of the neurite extension (yellow box in middle panel). The obvious markers (cell body, red arrows) were used to coordinate between the images and yellow box denotes the varicosity along the transfected neurites which was used to collect the tilt images.

In this study, we used NGF‐differentiated PC12 cells combined with cryo‐ET to investigate the protein organization under docked SV (Fig. S1). Before differentiation, the PC12 cells contain a mixture of secretory vesicles, mainly consisting of ‘dense‐core’ vesicles (~ 100–150 nm in diameter) but also a second population of smaller ‘clear’ vesicles (~ 40–60 nm in diameter), which are similar to SV in composition and function 34, 35. NGF treatment (3–7 days) dramatically increased the relative abundance of the small, synaptic‐like vesicles (~ 45 nm in diameter), with a majority localizing to the neurite‐like varicosities that develop during differentiation (Fig. 1A,B). Importantly, these neurite varicosities were sufficiently thin and ideal for cryo‐ET analysis (Fig. 1C).

To preserve these neurite varicosities in their native state, we carried out an *in situ* vitrification, using NGF‐differentiated PC12 cells grown directly on gold EM grids. The NGF‐differentiated PC12 cells lack mature active zones and do not maintain a pool of release‐ready vesicles 34, 35. Thus, to increase the population of such vesicles under these conditions, we overexpressed a VAMP2 protein with mutations in the C‐terminal half (L70D, A74R, A81D & L84D; termed VAMP2‐4X), which allows SNAREpins to zipper approximately halfway but not completely, and as such allows vesicles to dock but not fuse 20, 21. EM images confirmed that the VAMP2‐4X overexpression does not alter the ultrastructure of the neurite varicosities as they were indistinguishable from the nontransfected neurites (data not shown). However, the proportion of SVs proximal to PM in VAMP2‐4X varicosities was nearly double that of nontransfected boutons (49.1 ± 0.03% vs 25.3 ± 0.03%, respectively, *P* < 0.05). Specifically, we used a version of VAMP2‐4X that was tagged with pHluorin on the luminal side of the SV and employed a correlative light‐EM (CLEM) workflow to identify the mutant neurite varicosities for cryo‐ET data collection (Fig. 1C).

**Figure 2:**
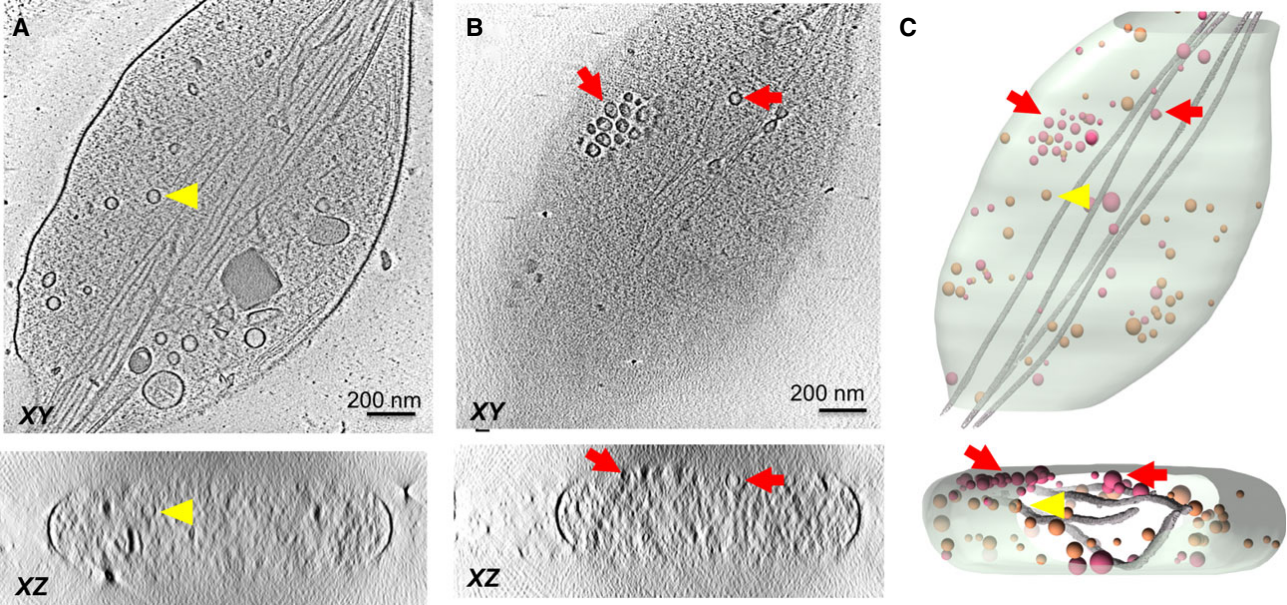
Representative tomographic slices and 3D segmentation rendering of NGF‐induced PC12 neurite varicosities shows the distribution of vesicles within the varicosities. Tomographic slices of VAMP2‐4X‐pHluorin‐transfected neurite varicosities both at (A) center of the varicosity and (B) proximal to the PM are shown (C) Segmentation of the neurite varicosity built from the tomographic density volume in (A and B). Only vesicles that locate proximal to the PM (red arrowhead/pink vesicles) were included in our analysis, while vesicles floating inside the varicosity (yellow arrowhead/orange vesicles) were excluded.

We acquired a tilt‐series using a 300‐kV Titan Krios; which along with the combination of direct electron detector and Volta‐phase plate and electron energy filter, resulted in tomograms with good contrast. To delineate the molecular architecture at the vesicle–membrane interface, we focused on the vesicles that locate close/proximal to the top or bottom PM (Fig. 2). We used the *XZ* slice of the tomographic 3D reconstruction to initially screen and identify PM‐proximal vesicles. For this subset of vesicles, we further carried out *Z*‐stack analysis on the *XY* slices and the absence of any organelles between the periphery of the vesicle and the boundary of the cell was used as the criteria to define ‘docked’ vesicles.

From the 574 high‐quality tomograms of neurites thus obtained, we generated reconstructions of 4757 docked vesicles. These vesicles were first sorted into twenty 3D‐classes based on the size and shape of the vesicles (Fig. S3). The 3D classes featuring spherical vesicles with diameter of 45 ± 5 nm were then selected to generate a homogeneous dataset. These were further subjected to several cycles of alignment and global averaging (using vesicle center as reference) until there was no further improvement in the homogeneity in size and shape (45 ± 2 nm). These vesicles (2434 in total) were pooled together for local alignment and averaging with the center of alignment now at the site of docking (i.e., SV–PM interface).

**Figure 3:**
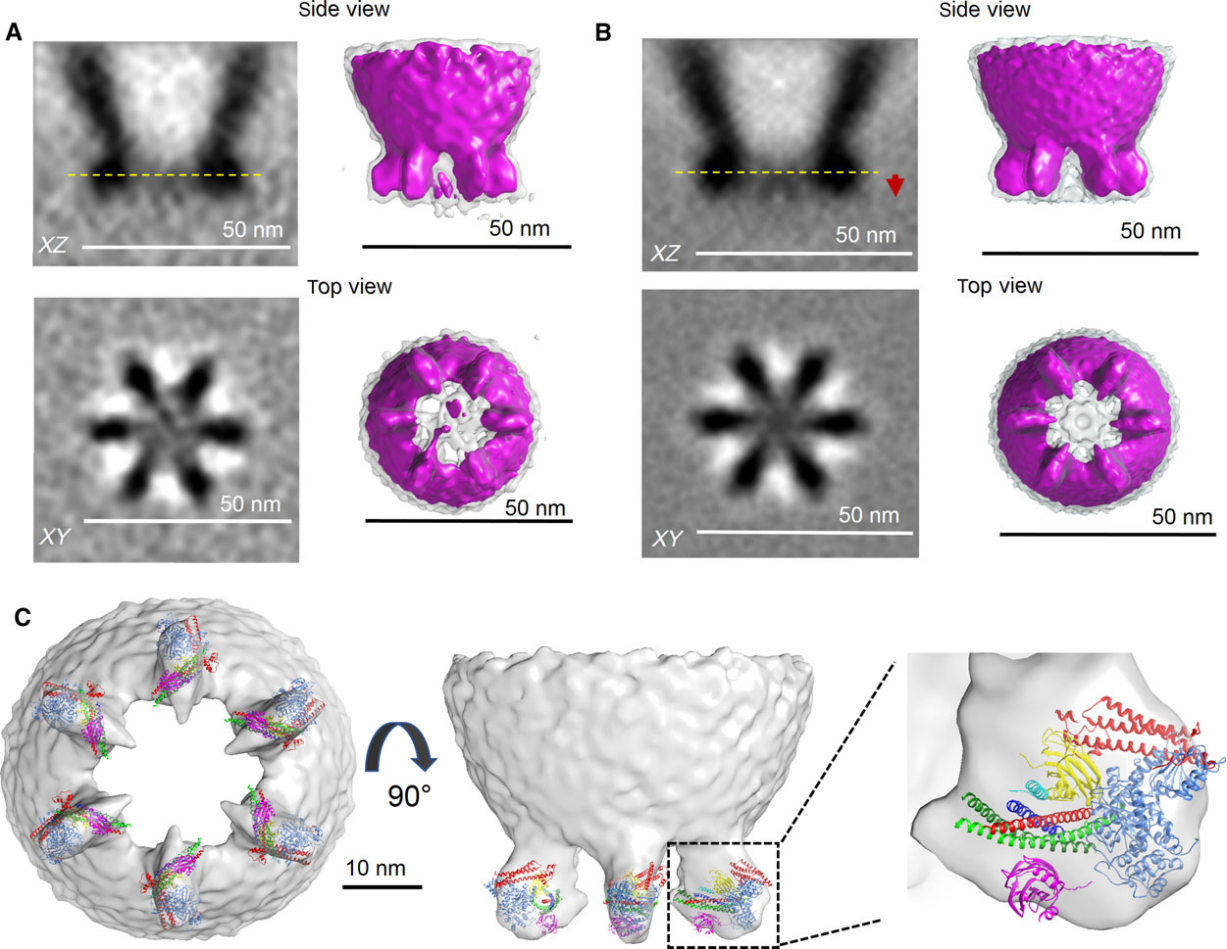
Cryo‐ET 3D reconstruction of the protein organization at vesicle‐ PM interface. 3D‐reconstructions both without (A) and with sixfold rotational symmetry imposed (B) reveals six symmetrically organized protein density under a docked vesicle. Left panels show the slices through the reconstructed volume of the averaged subtomograms. Top: slice along *z*‐axis. Red arrow points to relative position of the PM. Bottom: slice through the volume in *XY* plane at the vertical position highlighted by yellow dotted line on the top. Right panel shows the surface representation of the 3D map filtered to 36 Å at σ = 2 (magenta) and σ = 1 (transparent gray) threshold levels. (C) Rigid body fitting of crystal structures of constituent proteins into the densities below the vesicle. (A) Given the relatively low resolution, it is not possible to rigorously assign specific domains to features in the 3D map, but each of the pronounced density observed between the vesicle and PM were best fitted with a protein complex, consisting of SNAP (SNAP‐25 – green, Syntaxin‐1A – red, VAMP2 – blue); Cpx (cyan), two Synaptotagmin C2B molecules – primary (magenta) and tripartite (yellow) and Munc18 (light blue). Note: Fitting into the sixfold rotationally symmetrized cryo‐ET map at threshold level σ = 2 is shown and Syt1 C2A domains are omitted for clarity. The following X‐ray structures (PDB code) 5W5C (Syt1‐Cpx‐Syt1); 4JEU (Munc‐18/Syntaxin‐1 Habc domain complex) were manually fitted into the cryo‐ET map using USCF Chimera software.

After five cycles of alignment and classification, the tomograms were sorted into ten 3D‐classes (Fig. S3). In each and every 3D‐class, we observed six rod‐like densities in the *XY* plane corresponding to the vesicle–cell membrane interface. These protein densities were symmetrically organized in a clear majority (~ 80%) of the 3D‐classes. To improve the signal‐to‐noise ratio, all subtomograms in these final ten classes were pooled together and averaged, both without (Fig. 3A) and with imposing a sixfold rotational symmetry (Fig. 3B). The resulting density map (for both symmetrized and nonsymmetrized reconstructions at resolution of 5 nm and 5.4 nm respectively) showed six pronounced protein densities symmetrically organized (~ 60° apart) as if equally spaced on a common circle of ~ 35 nm diameter. There was also a consistent, but weaker protein density in the center.

The surface representation of the resulting 3D‐map revealed that each of the six radially‐organized protein masses likely corresponds to highly structured, large protein complex as it is prominent even at the highest threshold level (σ = 2). In contrast, the central protein mass was weak and visible (Fig. 3A,B) only at a lower threshold (σ = 1), signifying unstructured regions. Critically, no strong electron density was observed on the vesicle outside the PM contact regions, indicating that the observed protein organization was specific to the vesicle–membrane interface. Note that the six‐sided arrangement in the center of Fig. 3B is present only at the lower threshold and most probably, an artifact due to the imposed symmetry.

The synaptic‐like vesicles that we have reconstructed are expected to be analogous to the synaptic vesicles at neuronal synapses 34, 35. In these docked vesicles, SNAREs are ?clamped’ in an approximately half‐zippered state, such that they can release rapidly following Ca^2+^‐influx. Consistent with this, (a) these vesicles are located in close proximity to the PM; (b) they contain the overexpressed VAMP2‐4X‐pHluorin, which prevent fusion as shown by CLEM; (c) the lumen of these SV is inaccessible to protons from the extracellular medium (Fig. S4).

From established biochemistry and structural biology, each SNAREpin is known to be bound within an ‘exocytosis module’ consisting minimally of two copies of Syt1, one copy of Cpx, and one copy of Munc18. The combined molecular weight of this module is ~ 250 kDa. It is likely that each of the six symmetrical masses we have discovered consists of one such module. Modeling shows that the shape and size of each such mass is sufficient to accommodate one exocytosis module (Fig. 3C) but not two. At the present resolution, largely limited by the geometry of docked vesicle (only top/bottom and no side view) and the missing‐wedge effect, it is not possible to rigorously assign specific protein domains to features, even consistent features, within the masses. As a result of the missing wedge, the resolution in the *XY* plane is better than it is along the *Z* axis (perpendicular to the PM plane), which has the effect of exaggerating the shape of objects along *Z*. This likely explains why there appears to be significant unfilled space in *XZ* views (Fig. 3C, middle) but much less so in *XY* views (Fig. 3C, left). Altogether it is evident that the shapes are well occupied by one module and clearly cannot accommodate two modules. Therefore, we can clearly conclude that exactly six SNAREpins are present and likely co‐operate to release synaptic‐like vesicles (Fig. 3C).

How they co‐operate is not apparent from these images, but their exactly symmetrical positioning every 60° along a circle of ~ 35 nm outer diameter strongly suggests they are attached at regular intervals to an underlying framework. Considering that Syt1 is known to polymerize into ring‐like oligomers of similar diameters 11, 12, 13, and also to bind SNAREpins 15, the simplest hypothesis would be that each mass consists of a SNAREpin with its associated proteins bound to a Syt1 ring at regular intervals. We would not expect to directly visualize the proposed Syt1 ring at the present resolution if it were present due to its small thickness (~ 4 nm). The same is true for the even thinner MUN domain‐based outer rings predicted by the buttressed ring hypothesis.

**Figure 4:**
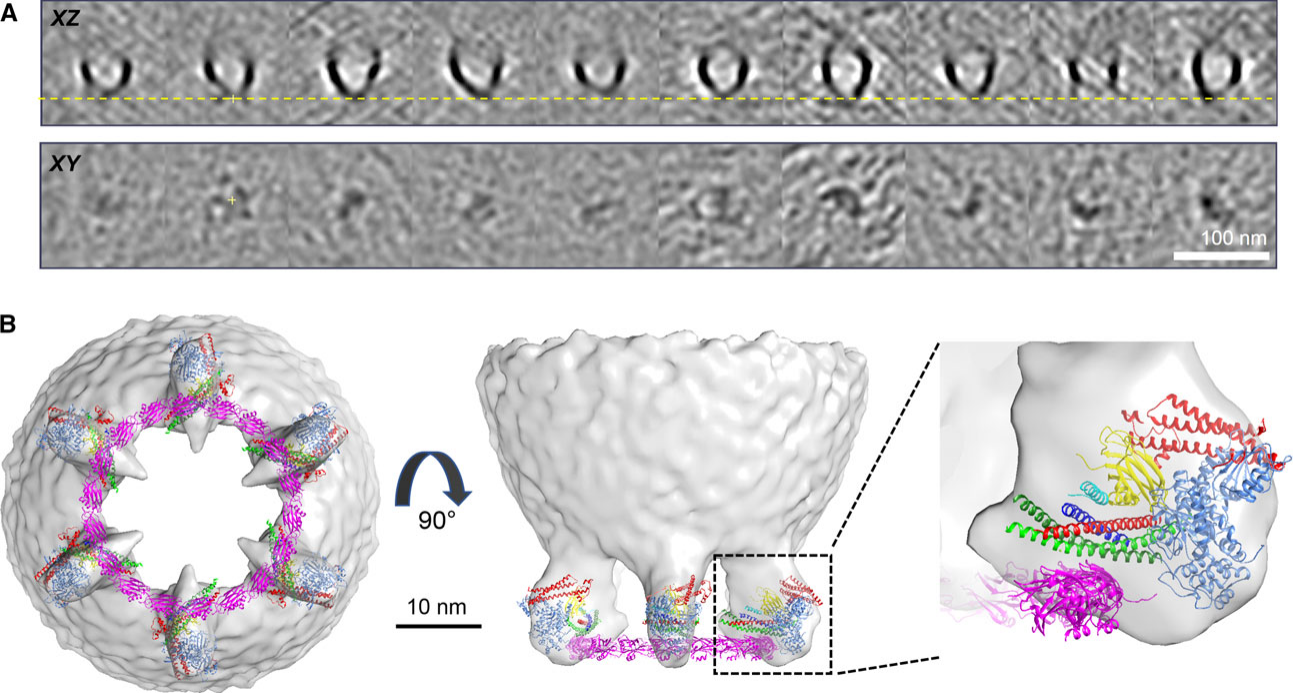
Protein organization at the vesicle‐membrane interface is templated by Synaptotagmin‐ring like oligomers. (A) Subtomogram class averages of docked vesicles in the PC12 cells expressing Syt1 oligomerization mutant (F349A) shows weak protein density, but lacks organization at the vesicle–PM interface. Top row: Slice through the center of tomogram along *z* axis. Bottom row is the corresponding slices through the volume in *XY* plane at the vertical position highlighted by yellow dotted line on the top panel. (B) Modeling shows how Syt1 ring‐like oligomers containing 18 Syt1 molecules are perfectly positioned to template the observed symmetrical organization of the exocytic modules. The six SNAREpins, bound to the regulatory proteins, can be positioned on top of the Syt1 oligomer bound *via* the ‘primary’ binding site. In addition to the X‐ray structures 5W5C (Syt1‐Cpx‐Syt1); and 4JEU (Munc‐18/Syntaxin‐1 Habc domain complex), the Syt1 oligomer model from Wang *et al*. 11 was used in the model building process using USCF Chimera software.

To test this hypothesis, we tested the effect of a Syt1 mutant (F349A), which is designed to specifically destabilize the Syt1 oligomers 14. As reported earlier, this F349A mutation supports vesicle docking and fusion in PC12 cells, but failed to clamp the vesicles 14. We over‐expressed the Syt1 F349A mutant, along with VAMP2‐4X‐pHluorin and generated 3105 reconstructions of docked SVs from 141 tomograms. We carried out subtomogram averaging and 3D classification exactly as before. We observed protein densities at the vesicle–membrane interface in the subtomogram class averages (Fig. 4A) but there was no evidence of symmetrical organization. In stark contrast, symmetrical organization was clearly evident in each of the subtomogram class averages for the wild‐type Syt1 (Fig. S3). We suspect that the observed densities with the F349A mutant are weak as the electron density of the dis‐organized exocytosis modules is smeared out during the averaging process. At the very least, the results establish that the same surface of the C2B domain that is utilized for ring‐like oligomer assembly *in vitro* is also needed to circularly organize the SNAREpin‐containing exocytosis modules, thus providing compelling albeit indirect evidence that the organizing framework consists of ring‐like oligomers of Syt1 (Fig. 4B).

How can precisely six SNAREpins be assembled from each SV when there is a very much larger supply of both v‐ and t‐SNAREs locally available? Evidently the limiting component must be a required chaperone. Of the two chaperones (Munc18 and Munc13) required for SNAREpin assembly 2, 3, 36, Munc18 enters the reaction bound to Syntaxin in a 1 : 1 stoichiometry 3, 36, 37 and thus, unlikely to be a limiting component. By elimination, this suggests that Munc13, which co‐operates with Munc18 to assemble half‐zippered SNAREpins, but enters separately is most likely the limiting component.

In formulating the buttressed ring hypothesis 16, we pointed out that the conserved MUN domain of each Munc13 forms an arc that subtends an angle of ~ 60° and thus can be naturally arranged to form a planar, closed circle formed of exactly six copies of these chaperones. This proposed outer ring of Munc13 can snugly enclose an inner ring of Syt1 oligomers and was predicted to assemble exactly six SNAREpins under each vesicle positioned every 60°, which would explain what we have now observed. We note that numerous related chaperones containing the conserved MUN domain of Munc13 play an essential role in the assembly of most if not all SNARE complexes, including those facilitating the majority of intracellular transport processes which occur constitutively, not involving a Synaptotagmin clamp 16, 38, 39. Perhaps these MUN domains also form rings and template six SNAREpins, which in the absence of an inner Synaptotagmin ring can proceed to fuse spontaneously without an imposed delay.

It may seem surprising that a handful of SNAREpins – if they are synchronously released – could achieve sub‐millisecond fusion pore opening. However, recent modeling that includes the concept of mechanical coupling predicts exactly this 40. Membranes are rigid on the length scale of a fusion pore, and as a result, SNAREpins will necessarily be mechanically coupled. Each time any one of the SNAREpin zippers up, it pulls the membranes closer together, triggering its neighbors to zipper at nearly the same time 40. At this stage, each SNAREpin still has ~ 5 k_B_T of mechanical work to deliver toward overcoming the bilayer fusion barrier (~ 25 k_B_T for physiologically relevant lipid compositions 41). For a single SNAREpin, this barrier is overcome in about 1 s 4, 5, 6 Therefore, each added SNAREpin reduces this waiting time for fusion by a factor of about 10^2^ (calculated as *e* ^(−5 kBT)^), so as the extraordinary consequence of mechanical coupling just three SNAREpins will reduce the time for vesicle release to ~ 0.1 ms 40.

Another feature of mechanical coupling is that, together with the measured energy landscape of the SNAREpin zippering 42, it also predicts that there is an optimum number of SNAREpins that maximizes fusion rate 40. The reason is that fusion cannot occur until the last SNAREpin fully zippers, and when there are a large number of them this becomes rate limiting. Each SNAREpin is metastable in the half‐zippered conformation 42, stabilized by a small energy well resulting from the polar ‘zero’ layer of the four‐helix bundle that interrupts the otherwise continuous hydrophobic heptad repeat 43. Though small for any one of the SNAREpin, the probability that one or more SNAREpins are ‘left behind’ increases exponentially with the number of SNAREpins 40. The tradeoff between these opposing processes predicts an optimum of 4–6 SNAREpins is required to achieve submillisecond release as needed for synaptic transmission, depending on the exact choice of various parameters 40. It is remarkable that this optimum, predicted from first principles, should align so closely with what molecular imaging of synaptic‐like vesicles *in situ* suggests.

## Author contributions

XL, AR designed experiments, collected, analyzed and interpreted cryoET data. KG analyzed/interpreted cryoET data and built molecular models. RK, AC and SG optimized cell cultures, over‐expression and collected data. SSK, JL and JER designed the experiments, provided supervision, analyzed/interpreted the data and wrote the manuscript. All authors read and revised the manuscript.

## Supporting information


**Fig. S1**. Work‐flow for Cryo‐CLEM and Cryo‐ET imaging of NGF‐differentiated PC12 cells.
**Fig. S2.** Western blot analysis of whole cell lysate shows that VAMP2‐4X is at least 20‐fold overexpressed as compared to the endogenous VAMP2.
**Fig. S3**. Flow chart for the cryo‐ET analysis used to obtain the protein organization at the vesicle‐PM interface.
**Fig. S4**. Ammonium chloride treatment shows majority of the vesicles in NGF‐differentiated PC12 cell neurites are docked but unfused.
