# Supplementary material for: Symmetrical organization of proteins under docked synaptic vesicles
Source: FEBS Lett. 2019 Jan 18;593(2):144–53. doi: 10.1002/1873-3468.13316 (PMC6353562; doi:10.1002/1873-3468.13316)
Supplement: Supplementary file 1 — Fig. S1. Work‐flow for Cryo‐CLEM and Cryo‐ET imaging of NGF‐differentiated PC12 cells. Fig. S2. Western blot analysis of whole cell lysate shows that VAMP2‐4X is at least 20‐fold overexpressed as compared to the endogenous VAMP2. Fig. S3. Flow chart for the cryo‐ET analysis used to obtain the protein organization at the vesicle‐PM interface. Fig. S4. Ammonium chloride treatment shows majority of the vesicles in NGF‐differentiated PC12 cell neurites are docked but unfused. [file FEB2-593-144-s001.docx]

Supplementary Information

Symmetrical Organization of Proteins Under Docked Synaptic-Vesicles

Xia Li^1,4*^, Abhijith Radhakrishnan^2,*^, Kirill Grushin^2^, Ravi Kasula^2^, Arunima Chaudhuri^2^, Sujatha Gomathinayagam^2^, Shyam S. Krishnakumar^2,3^, Jun Liu^1,2,#^ and James E. Rothman^2,3,#^

^1^Department of Microbial Pathogenesis, ^2^Department of Cell Biology, Yale University School of Medicine, New Haven, CT 06520, USA.^3^Department of Clinical and Experimental Epilepsy, UCL Queen Square Institute of Neurology, London, WC1 3BG, UK. ^4^Institute of Nautical Medicine, Co-innovation Center of Neuroregeneration, Nantong University, Nantong, Jiangsu 226001, China

*These authors contributed equally

^#^*Correspondence*: james.rothman@yale.edu; jliu@yale.edu.


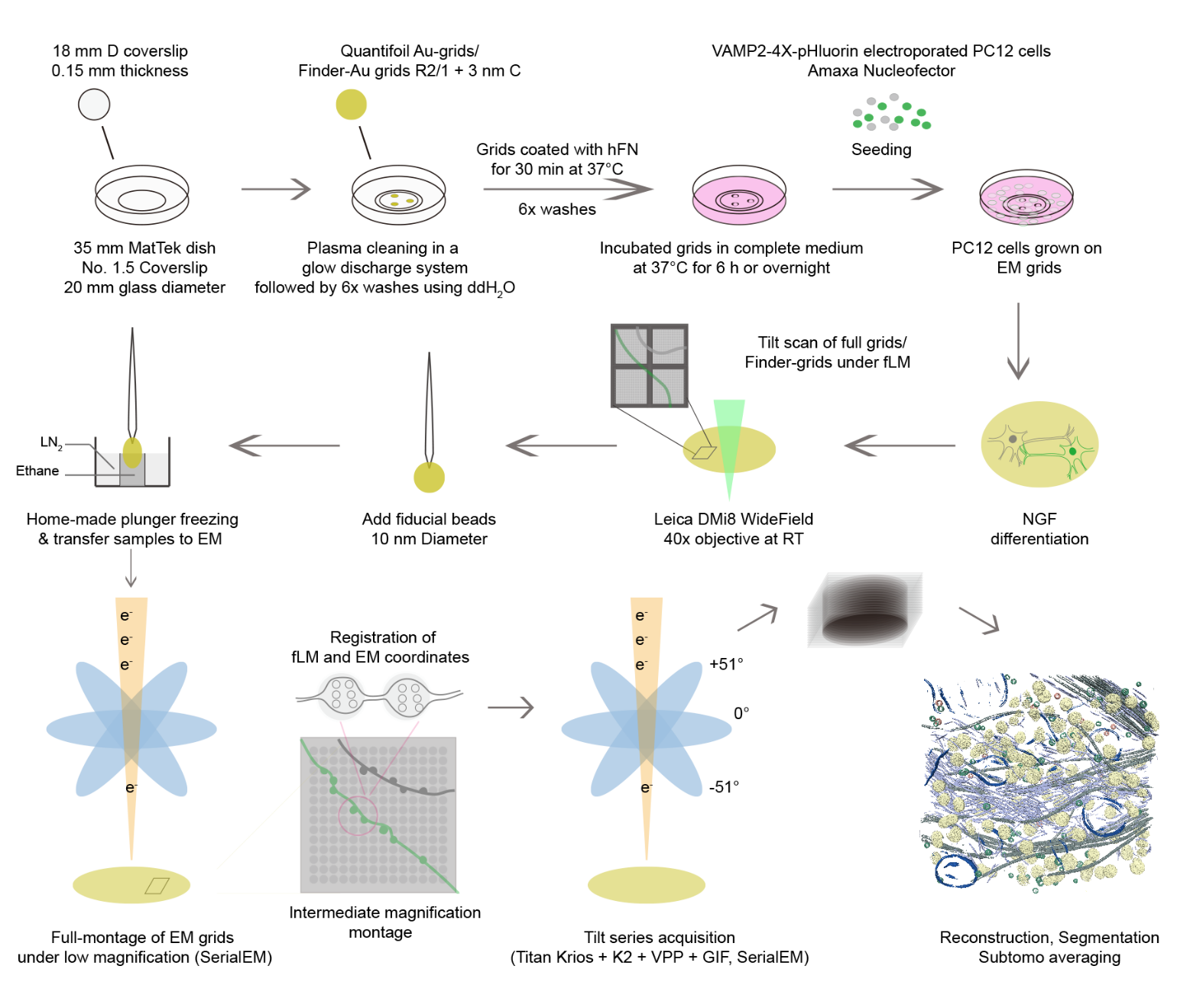


**Figure S1**. Work-flow for Cryo-CLEM and Cryo-ET Imaging of NGF-differentiated PC12 cells. NGF differentiated PC12 cells transfected with VAMP2-4X-pHluorin are transferred to pre-treated gold EM grids and grown for 7 days. The specimen was evaluated by fluorescence light microscopy (fLM) for locating the transfected cells and then were plunge-frozen with a homemade plunger. The frozen grids are imaged on Titan Krios to obtain a full-montage recorded at 220X using SerialEM software. The cryo-EM montage and fLM maps were merged together before acquiring intermediate magnification montages at 3600X for identifying the most suitable positions for tilt series data collection. The dataset was then reconstructed and further processed by segmentation, measurement analysis, and sub-tomogram averaging analysis.

**
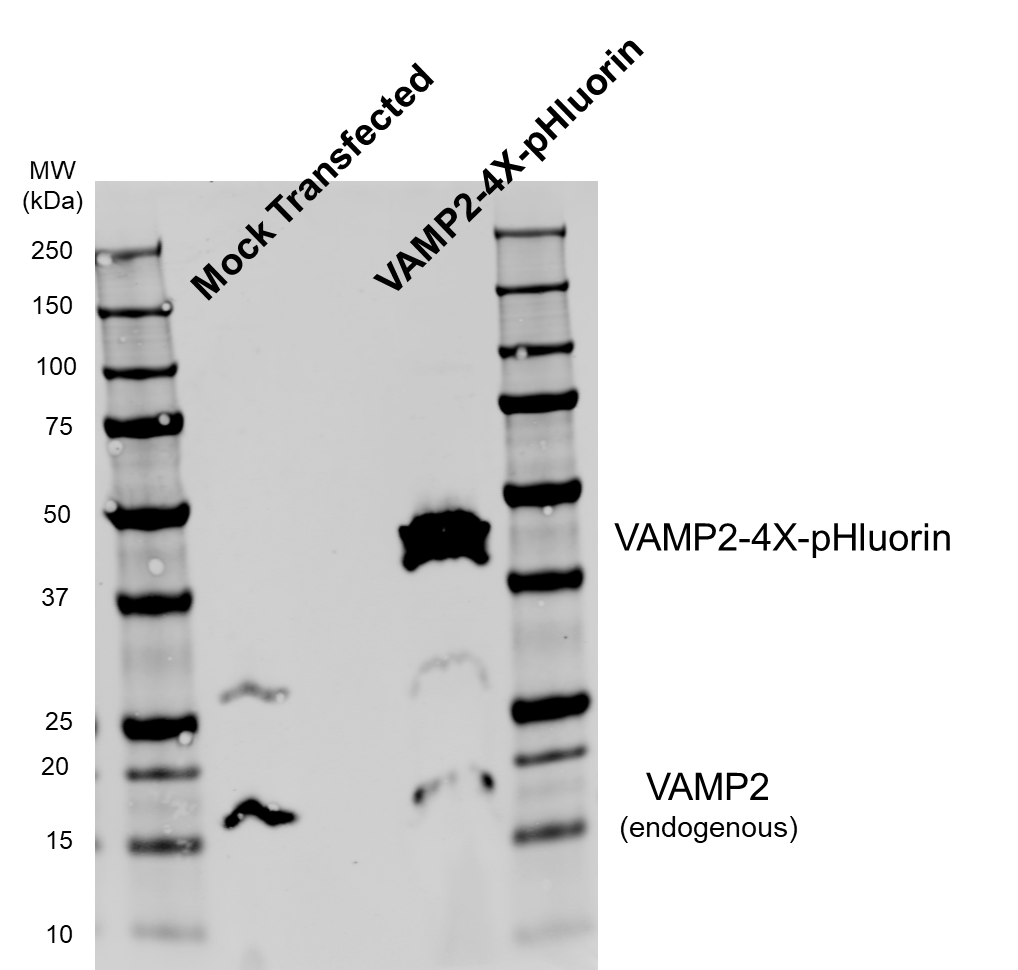
**

**Figure S2.** Western blot analysis of whole cell lysate shows that VAMP2-4X is at least 20-fold overexpressed as compared to the endogenous VAMP2. PC12 cells were grown in six-well plates coated with fibronectin. After 48h of transfection, cells were washed, detached, and collected by centrifugation. Pellets were re-suspended in radio-immunoprecipitation assay (RIPA) buffer for 1 h followed by spin down at top speed (20,817g) in a table top centrifuge for 10 min. Protein estimation in the post-nuclear cell lysates were performed using Bicinchoninic acid (BCA) assay reagent from Pierce (Rockford, IL). Equal quantity (~10 µg) of protein from both the cell lysates were first separated in SDS-PAGE and then transferred onto PVDF membranes (Millipore, Billerica, MA) for immune-blotting. After being blocked with Odyssey Blocking Buffer (LI-COR Biosciences, Lincoln, NE, USA), the membranes were incubated with antibody against VAMP2 (synaptic systems cat #104 211). The levels of individual proteins were determined by quantitative Western blotting with IRDye conjugated secondary antibodies (IRDye® 680RD Goat anti-Mouse IgG) on a LI-COR infrared imaging system. Fluorographs were quantitatively scanned using ImageStudio and quantitative analysis of the western blot showed ~ 20-fold overexpression of VAMP2-4X-pHlourin over endogenous VAMP2. Representative immunoblot is shown.


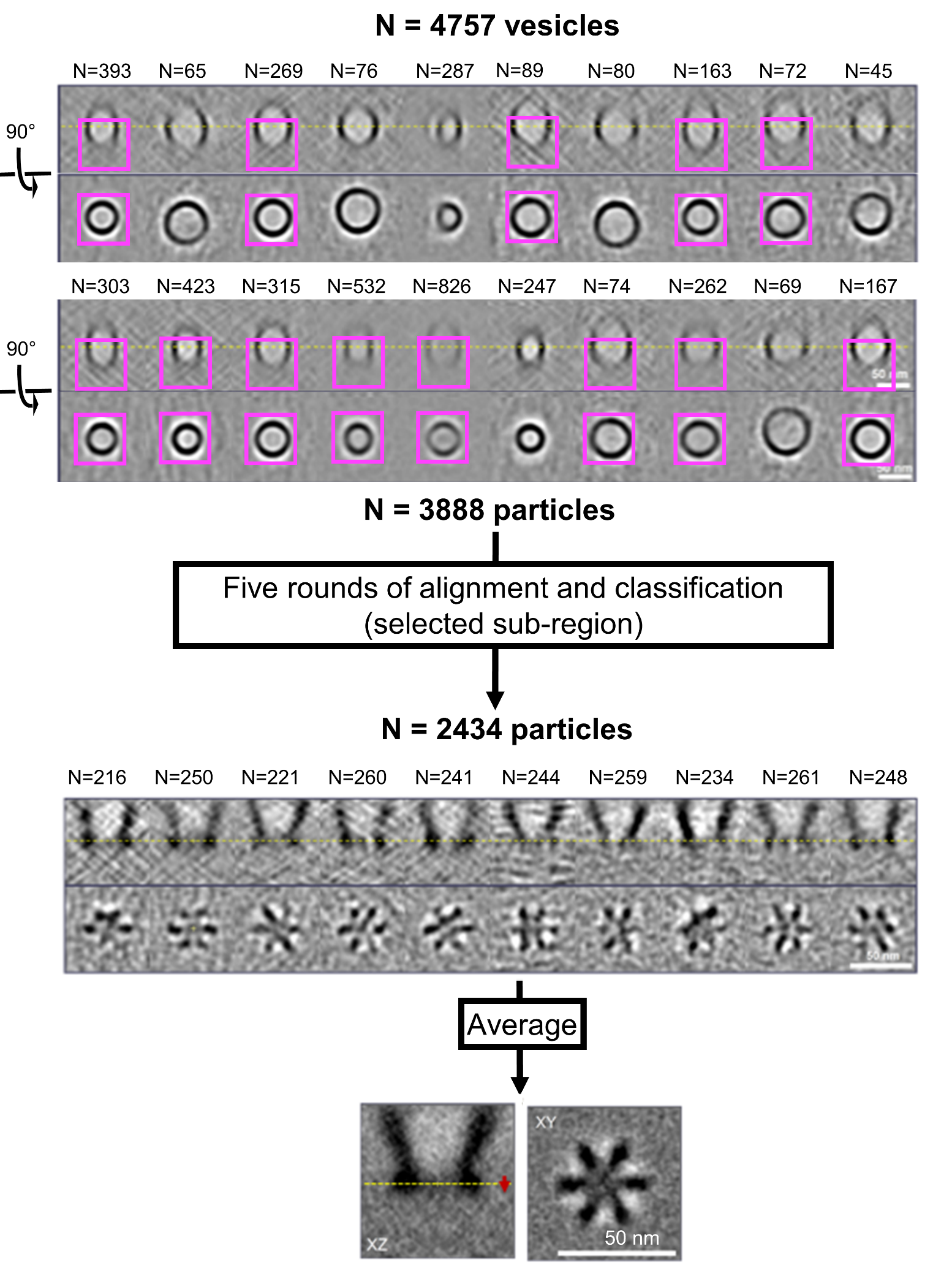


**Figure S3**. Flow chart for the cryo-ET analysis used to obtain the protein organization at the vesicle-plasma membrane interface. Sub-tomogram class averages of all the selected docked vesicles (4757 vesicles) were initially aligned based on the size and shape of the vesicles were sorted into 20 classes (top panel). The classes containing vesicles sized 45 ± 5 nm were selected for further processing (magenta boxes in top panel). These sub-set of vesicles (3888 vesicles) were subjected to additional rounds of alignment and classification until no further improvement was observed. The resulting vesicles (2434 vesicles) were then used for local alignment to identify the protein density at the site of docking. 3D reconstruction of the docked vesicles without imposed symmetry classified into ten 3D-classes is shown in the middle panel. It revealed six prominent rod-like densities arranged radially. The ten classes were averaged to produce the final 3D reconstruction (bottom panel). In all cases, slice through the center of tomogram along Z-axis and the corresponding slices through the volume in XY plane at the vertical position highlighted by yellow dotted line on the top panel are shown. Scale bar is 50 nm.


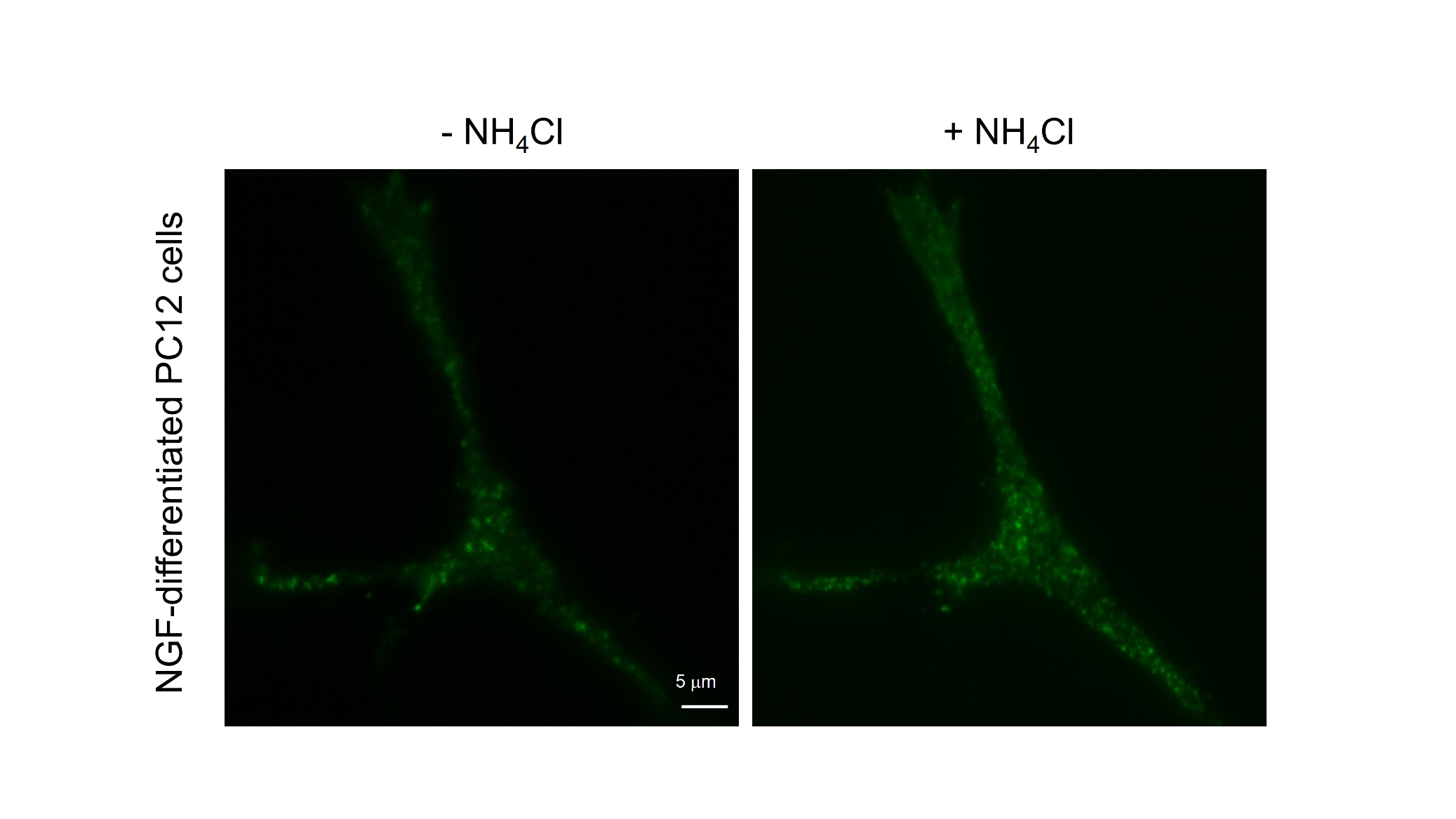


**Figure S4**. Ammonium chloride treatment shows majority of the vesicles in NGF-differentiated PC12 cell neurites are docked but unfused. Fluorescence imaging of pHluorin-VAMP2-4X (luminal tag) under total internal reflection fluorescence (TIRF) conditions show that the majority of the vesicles near/at the plasma membrane of the PC12 neurites are non-fluorescent under control conditions (left panel), but become strongly fluorescent following neutralizing ammonium chloride (NH_4_Cl) treatment. As the pHluorin fluorescence is pH sensitive and is quenched under low pH conditions, this shows that in a majority of the docked vesicles, the vesicle lumen (i.e. low pH condition) is intact corresponding to an un-fused state. PC12 cells were transfected with VAMP2-4X-pHlourin using electroporation and platted on fibronectin coated mattek dishes and incubated in DMEM culture media. Fresh DMEM containing NGF was changed on day 3 of transfection for neurite growth. On day 7, 1000 frames of PC12 cell neurite were captured on TIRF plane at 100 ms per frame in live imaging buffer (140 mM NaCl, 2.5 mM KCl, 1.8 mM CaCl_2_, 1 mM MgCl_2_). To minimize the background from pHluorin signal on the plasma membrane and to unambiguously track vesicles under the control conditions, the cells were treated with acidic 2-(N-morpholino)ethanesulfonic acid (MES) buffer (15 mM, pH 5.0) right before imaging. The pH of the medium was subsequently neutralized using NH_4_Cl (100 mM) to visualize all docked vesicles. Representative fluorescent images of neurites ~1 sec post treatment is shown in both cases.
